# Effects of Dietary Supplementation of Garlic and Oregano Essential Oil on Biomarkers of Oxidative Status, Stress and Inflammation in Postweaning Piglets

**DOI:** 10.3390/ani10112093

**Published:** 2020-11-11

**Authors:** Jorge Rivera-Gomis, Camila Peres Rubio, Cristina Martínez Conesa, Julio Otal Salaverri, José Joaquín Cerón, Damián Escribano Tortosa, María José Cubero Pablo

**Affiliations:** 1 Research Group E095-06 Antimicrobial Resistance in Animal Health, Regional Campus of International Excellence “Campus Mare Nostrum”, University of Murcia, 30100 Espinardo, Murcia, Spain; jorge.rivera@um.es (J.R.-G.); mjcubero@um.es (M.J.C.P.); 2 Interdisciplinary Laboratory of Clinical Analysis of the University of Murcia (Interlab-UMU), Regional Campus of International Excellence “Campus Mare Nostrum”, University of Murcia, 30100 Espinardo, Murcia, Spain; camila.peres@um.es (C.P.R.); jjceron@um.es (J.J.C.); 3 Research Group on Rainfed Agriculture for Rural Development, Department of Rural Development, Oenology and Sustainable Agriculture, Murcia Institute of Agri-Food Research and Development (IMIDA), 30150 Alberca Las Torres, Murcia, Spain; cristina.martinez4@carm.es; 4 Animal Production Department, Regional Campus of International Excellence “Campus Mare Nostrum”, University of Murcia, 30100 Espinardo, Murcia, Spain; juotal@um.es

**Keywords:** micro capsuled oregano essential oil, weaned piglets, purple garlic powder, biomarkers, pig production, oxidative status

## Abstract

**Simple Summary:**

The effects of two different concentrations of micro capsuled oregano essential oil (OEO) and purple garlic powder on biomarkers of oxidative status, stress, and inflammation, as well as on average daily gain (ADG) and feed conversion ratio (FCR), were evaluated in piglets during the postweaning period. The trial was carried out with 300 crossbred pigs of 21 days of age fed with different concentrations of OEO and purple garlic powder and ZnO. Saliva and serum samples were taken to evaluate a panel of biomarkers of oxidative status, stress, and inflammation. OEO and garlic powder at 0.4% did not produce significant changes in C-reactive protein (CRP) and cortisol and yielded higher levels of the antioxidant biomarker CUPRAC in serum than higher doses (*p* < 0.01); they also yielded a better ADG than the control and ZnO diets. OEO and garlic powder at higher concentrations than 0.4% showed higher concentrations of CRP (*p* < 0.05). Overall, doses of OEO and garlic powder at 0.4% did not lead to inflammation, stress, or negative changes in oxidative biomarkers in piglets during the postweaning period and gave better productive performance than the control and ZnO diets. High doses of OEO and garlic powder were ineffective.

**Abstract:**

The effects of two different concentrations of micro capsuled oregano essential oil (OEO) and purple garlic powder on biomarkers of oxidative status, stress, and inflammation, as well as on average daily gain (ADG) and feed conversion ratio (FCR), were evaluated in piglets during the postweaning period. The trial was carried out with 300 crossbred pigs of 21 days of age fed with different concentrations of OEO and purple garlic powder and ZnO. Saliva and serum samples were taken to evaluate a panel of biomarkers of oxidative status, stress, and inflammation. OEO and garlic powder at 0.4% did not produce significant changes in C-reactive protein (CRP) and cortisol and yielded higher levels of the antioxidant biomarker CUPRAC in serum than higher doses (*p* < 0.01); they yielded a better ADG than the control and ZnO diets. OEO and garlic powder at higher concentrations than 0.4% showed higher concentrations of CRP (*p* < 0.05). Overall, doses of OEO and garlic powder at 0.4% did not lead to inflammation, stress, or negative changes in oxidative biomarkers in piglets during the postweaning period and gave better productive performance than the control and ZnO diets. High doses of OEO and garlic powder were ineffective and could negatively affect the animals. Therefore, our results highlight the importance of the dose used when OEO or garlic are supplemented to piglets.

## 1. Introduction

Weaning is a critical period in pig production that determines the productive performance of commercial farms. Practices such as mixing and changes in nutrition and environment can negatively affect the endocrine functions, growth, and welfare of weaned piglets [1,2]. Therefore, weaning is a stressful period that produces increases in salivary stress markers such as cortisol [3,4,5]. It also induces changes in oxidative stress markers and intestinal dysfunctions [6]. In addition, mucosal inflammation, digestive performance, and body weight gain are also affected by early weaning [7,8,9].

In order to reduce the impact of the abovementioned issues, therapeutic dietary addition of zinc as zinc oxide (ZnO) (2000 to 4000 mg/kg) has been widely used during the postweaning period, being effective in reducing diarrhoea and digestive dysfunctions [9,10,11,12,13]. ZnO improves postweaning performance and reduces the effects of postweaning colibacillosis by inhibiting cAMP-stimulated chloride secretion [14]. Bactericidal activity of ZnO has been observed in vitro, and, therefore, therapeutic levels of ZnO may reduce enterotoxigenic *Escherichia coli* (ETEC) colonization and bacterial population in the intestine [15]. Nevertheless, the use of ZnO at therapeutic levels has serious drawbacks such as environmental pollution due to the high proportion excreted in faeces [9,16,17] and its possible association with antimicrobial resistance [18,19]. These facts have led to the prohibition of its use at high doses from 2022 in the European Union (EU) [20].

Due to the problems surrounding the use of ZnO and its future restriction, research to find economically and environmentally friendly alternatives is being carried out in order to contribute to the sustainability and resilience of the pig production sector. Plant extracts are widely used as feed additives to enhance the productive performance of several animal species and represent a potential alternative to ZnO due to their properties [21]. Some of the plant extracts that could be used as alternatives are oregano (*Origanum vulgare* L.) and garlic (*Allium sativum*).

Oregano contains high concentrations of carvacrol and thymol (around 80%) [22] that have demonstrated, in vitro, significant antimicrobial, antifungal, and antioxidant activity [23,24,25]. Furthermore, its use appears to improve growth performance in weaned pigs [26,27], as well as in other species such as growing lambs and broilers [28,29,30]. In addition, Zhang et al. [31] showed that oregano could reduce serum cortisol levels and increase antioxidant enzyme activity after transportation stress in pigs. However, other reports have indicated that oregano administration can cause cell damage [32]. Oregano essential oil (OEO) can be micro capsuled to improve its stability, preserve the volatile compounds until consumed by animals, and ease its handling and administration. Several materials, such as sunflower oil, are used for this purpose [33].

On the other hand, garlic (*Allium sativum*) has the beneficial effects of antibacterial, antiviral, antifungal, antioxidant, and immunomodulatory activities [34,35,36,37,38]. The active compounds in garlic that seem to have beneficial effects are sulphide breakdown products such as alliin, diallylsulphides, and allicin [34]. Several studies have shown that dietary supplementation of garlic appears to improve the immune response and growth performance of growing pigs [39,40,41]. Horn et al. [42] observed that this plant could partially mitigate the stress effects of the postweaning period, producing a positive effect on growth performance, intestinal function, and antioxidant status. In addition, a study in chickens showed a reduction of heat stress due to dietary supplementation of garlic [43].

Although serum is usually used to measure biomarkers that can provide information about oxidative status in pigs [44,45], saliva can also be used in this species [46] with the advantage of being a sample that can be easily collected without producing stress to the pigs.

Research has been carried out on the effect of supplementation with OEO on the oxidative status in plasma and muscles of growing-finishing pigs, in the semen of boars, and in porcine small intestinal epithelial (IPEC-J2) cells in vitro. The anti-inflammatory effect of OEO has been associated with decreased cellular levels of reactive oxygen species (ROS), biomarkers of stress, such as cortisol, and biomarkers of inflammation, such as acute-phase proteins [47,48,49,50,51,52]. We hypothesized that garlic powder and OEO as feed additives at appropriate doses could improve the oxidative status, stress, and inflammation in weaned piglets. The purpose of this study is to evaluate and to compare the effects of dietary supplementation of two different concentrations of garlic powder and OEO with the supplementation of ZnO at therapeutic doses during the postweaning period of piglets in farm conditions, evaluating the oxidative status, stress, and inflammation of the animals. For this objective, a panel of biomarkers of oxidative stress in serum and saliva, a biomarker of stress in saliva (cortisol), and a biomarker of inflammation in serum (C-reactive protein (CRP)) were evaluated.

A panel of various analytes involving antioxidant and oxidant biomarkers were used, as this kind of panels has been recommended in order to have thorough information on the oxidative status [53]. Therefore this panel was comprised of thiol, catalase, uric acid, Trolox equivalent antioxidant capacity (TEAC), cupric reducing antioxidant capacity (CUPRAC), and ferric reducing ability of plasma (FRAP) as biomarkers of the antioxidant response and total oxidant status (TOS), advanced oxidation protein products (AOPP), and hydrogen peroxide (H_2_O_2_) as oxidant biomarkers. These biomarkers were measured in both serum and saliva in order to assess the possible use of saliva as an alternative or complement to serum in oxidative status evaluation. In saliva, the same biomarkers as serum were measured except for thiol and TOS because, to the author’s knowledge, there are currently no methods that can accurately measure these biomarkers in pig saliva [46].

## 2. Materials and Methods

### 2.1. Additives and Feed Composition

ZnO was administered as Zincotrax (Andrés Pintaluba S.A., Tarragona, Spain) containing 1000 mg of ZnO/g. The final dose in the ZnO group was 3100 mg of ZnO per kg of feed (equivalent to 2500 mg of Zn per kg of feed).

The essential oils of *Origanum vulgare* L. were acquired from Esencias Martínez Lozano S.A (Murcia, Spain). The extraction system was by steam distillation in an industrial boiler. The composition of the essential oil is detailed in Table 1, according to an analysis performed by gas chromatography-mass spectrometry (GC–MS) analysis. Oregano oil (1 µL diluted 5% in hexane) was subjected to analysis by GC–MS. An Agilent HP6890 gas chromatograph (GC), equipped with an HP-INNOWax (60 m × 0.25 mm i.d.) and 0.5 µm film thickness, was used. The stationary phase was supplied by Agilent Technologies (Palo Alto, CA, USA). Helium was used as the carrier gas (constant pressure 23 psi), and the split ratio was set to 100:1. The GC was linked to an Agilent model 5972 inert mass spectrometry detector (Agilent Technologies, Palo Alto, CA, USA). The initial oven temperature was set at 60 °C for 6 min, increased at 80 °C at a rate of 2 °C/min, then increased to 120 °C at 1 °C/min, and finally raised to 250 °C at a rate of 4 °C/min; the port and the transfer line to the mass selective detector were kept at 250 and 280 °C, respectively. The mass spectrometer was operated in electron impact ionization mode with ionizing energy of 70 eV, scanning from m/z 30 to 350 at 3.21 scan/s. The quadrupole temperature was 150 °C, and the electron multiplier voltage was maintained at 1300 V. The individual peaks were identified by retention times and by comparison of mass spectra using the NIST 75 K library (US National Bureau of Standards, 2002) and spectra obtained from the standard. Percentage compositions of samples were calculated according to the area of the chromatographic peaks using the total ion current.

The encapsulation was carried out by the company AT CAPSELOS SL. (Huesca, Spain). The 10% essential oil was encapsulated in mono and diglyceride coverage of edible fatty acids (as a low hydrophile–lipophile balance (HLB) emulsifier) and hydrogenated sunflower fat at a size of 800 microns.

Purple garlic powder (Allibia Fresh Flour, Adibio S.L., Teruel, Spain; see Table 2) containing 63% of purple garlic in the form of mashed and dried powder, with citric acid and silicic acid (E-551) as additives, was added to the basal diet in the Garlic 2% group.

The composition of the control diet is shown in Table 3. It was a commercial basal diet formulated to meet or exceed the energy and other nutritional requirements for weaned piglets during the postweaning period [54]. The piglets received two diets: the first from 21 days of age until they reached 10 kg of weight (prestarter) and the second diet until the end of the trial at ten weeks of age (starter). Both diets were composed of the usual ingredients used in pig feed (Table 3). No antibiotic was included in the diets.

### 2.2. Animals, Housing, and Experimental Design

The Ethical Committee for Animal Experimentation (CEEA) of Murcia University approved all the experimental protocols used in this study (Authorization Code 471/2018). All animal handling protocols were carried out according to animal welfare legislation in force in the EU [55,56]. The trial was carried out from April to December 2019.

The animals were obtained from Dalland Hybrid España S.A. (Murcia, Spain) and kept on a commercial farm in field conditions. A total of 300 crossbred pigs (Pietrain × Large White × Landrace) were randomly allotted to one of 6 treatments and divided into 10 replicates, with five pigs per pen in each replication following weaning at 21 days of age. The initial body weight (BW) of the piglets was 5.65 ± 0.26 kg, and there were no significant differences in BW between groups at the start of the trial (*p* = 0.166, one-way ANOVA; Table 4). The same number of females and males were included in the study and sampling. The experimental unit for performance data analysis was the pen of 5 piglets.

The experimental unit for performance data analysis was the group of 50 piglets (25 females and 25 males in separated pens). The animals were under experimental conditions for 49 days. The prestarter phase lasted 23 days, and the second phase lasted 26 days. All groups had continuous *ad libitum* access to feed and water through nipple drinkers and feed troughs. At the end of the experiment, the animals were sent to a growing-finishing farm to continue their productive cycle under commercial conditions.

The dietary treatments were as follows: (1) control (basal diet; *n* = 50); (2) ZnO, containing 3100 mg/kg of ZnO added to the basal diet (*n* = 50); (3) micro capsuled OEO at a concentration of 0.4% in the basal diet (*n* = 50); (4) micro capsuled OEO at a concentration of 1.2% in the basal diet (*n* = 50); (5) garlic powder at a concentration of 0.4% in the basal diet (*n* = 50); (6) garlic powder at a concentration of 2% in the basal diet (*n* = 50).

### 2.3. Sample Collection and Productive Measures

At the end of the trial, blood and saliva samples were collected from 17 piglets of each group that were randomly selected, ensuring that the sample was evenly distributed between the replicates, so each replicate had at least one piglet sampled. Blood samples were collected from the jugular vein for analysis using a 5-mL vacuum test kit without anticoagulant (VACUTEST KIMA srl., Arzergrande, Padova, Italy) to obtain serum samples. Serum samples were separated within 2 h of collection by centrifugation for 5 min at 3500× *g*. Saliva samples were collected using Salivette^®^ tubes (Sarstedt, Aktiengesellschaft & Co. D97 51588 Nümbrecht, Germany) containing a sponge instead of a cotton swab. The piglets were allowed to chew on the sponge, which was clipped to a flexible thin metal rod, for 1–2 min until thoroughly moist. Tubes were kept on ice until arrival at the laboratory and then centrifuged at 3500× *g* and 4 °C for 10 min to obtain saliva. Samples were stored at −80 °C until the day of analysis. No saliva samples with the presence of food or any other contamination were included in the study.

Body weight was measured at the beginning and the end of the trial. Feed intake was registered for every group during the whole trial. Average daily gain (ADG) was calculated by dividing the mean increase in weight in the group by the number of days that the trial lasted. To calculate the feed conversion ratio (FCR), the amount of feed consumed was divided by the weight gained during the studied period for each group. Average feed intake (AFI) was calculated for each group by dividing the amount of feed consumed per group between the number of animals. Final body weight (FBW) was calculated as the mean final animal weight for each group.

### 2.4. Measurements of Biomarkers

#### 2.4.1. Oxidative Status

Serum thiol concentrations were measured according to the method described by Jocelyn [57] and modified by Costa et al. [58]. Uric acid was measured using a commercial kit (OSR6198, uric acid, Beckman Coulter Inc., Fullerton, CA, USA). Antioxidant activity of catalase in serum was assessed by an automatic method previously described by Slaughter and O’Brien [59].

Total antioxidant capacity of samples was assessed by three different methods: TEAC, CUPRAC, and FRAP. TEAC concentrations were evaluated according to the assay described by Arnao et al. [60], which is based on the inhibition of the radical ABTS by the sample. Serum CUPRAC was determined using a method based on the reduction of Cu2+ Cu1+ by the sample [61]. FRAP concentrations in serum and saliva (FRAS) were determined following the method of Benzie and Strain [62], which measures the ferric to ferrous ion reduction by the sample.

Serum TOS was measured by the method described by Erel [63]. AOPP in serum was determined according to a method described by Witko-Sarsat et al. [64]. H_2_O_2_ levels were analyzed using the assay described by Rhee et al. [65].

All assays in serum showed a between and within run imprecision that was lower than 15% and linear, with r > 0.9 after serial dilutions.

Uric acid, catalase, TEAC, CUPRAC, FRAS, AOPP, and H_2_O_2_ were also measured in saliva samples. Their measurements were based on previously validated methods for porcine saliva [46]. Assays were conducted on an Olympus AU400 automated chemistry analyzer (Olympus Diagnostica Europe GmbH, Ennis, Ireland).

#### 2.4.2. Salivary Cortisol

Salivary cortisol was quantified using an automated chemiluminescent immunoassay (Immulite 1000 cortisol, Siemens Medical Solutions Diagnostics, Los Angeles, CA, USA) that was previously validated for porcine saliva [66].

#### 2.4.3. Serum CRP

Serum CRP concentrations were determined with a commercial immunoturbidimetric assay (Beckman Coulter^®^, Inc., Fullerton, CA, USA) using an automated analyzer (Olympus AU600, Olympus Europe GmbH, Ennis, Ireland), as previously reported [67].

### 2.5. Statistical Analysis

Data analyses were performed using statistical procedures and software (GraphPad Software, San Diego, CA, USA). All data were evaluated for normality using the Shapiro–Wilk test. Regarding serum results, CRP, catalase, and TOS results in serum were not normally distributed; therefore, they were presented in median and interquartile range (IQR) values, and analysis of the results was made using a nonparametric Kruskal–Wallis test, followed by Dunn’s multiple comparisons test. Normally distributed data were presented as means ± standard deviation (SD) and compared using one-way ANOVA, followed by Tukey’s multiple comparisons test. Salivary results were not normally distributed and were compared using the Kruskal–Wallis test and Dunn’s multiple comparisons post-test.

Correlations between all the markers analyzed were determined using Spearman’s correlation analysis. A value of *p* < 0.05 was used to indicate significance in all analyses.

## 3. Results

### 3.1. Biomarkers of Oxidative Stress

**Figure 1:**
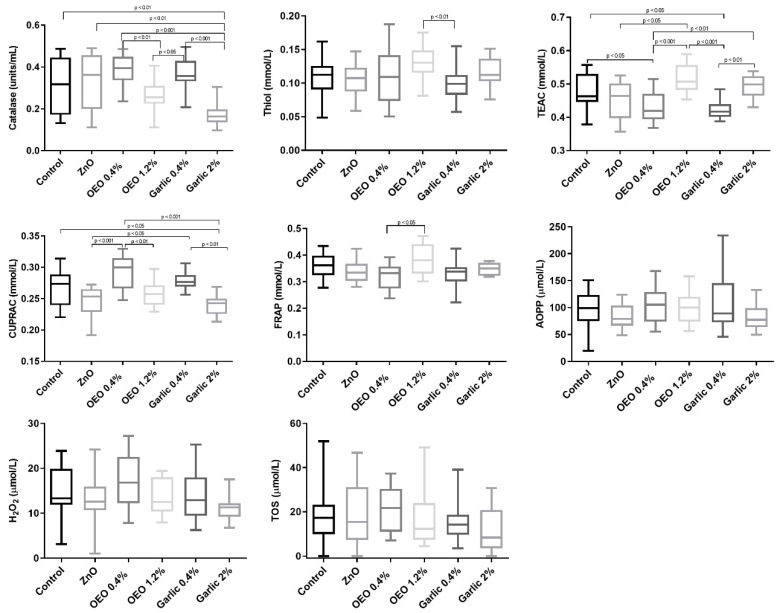
Results for serum catalase, thiol, Trolox equivalent antioxidant capacity (TEAC), cupric reducing antioxidant capacity (CUPRAC), ferric reducing ability of plasma (FRAP), advanced oxidation protein products (AOPP), hydrogen peroxide (H_2_O_2_), and total oxidant status (TOS) in piglets supplemented with ZnO, micro capsuled OEO at a concentration of 0.4% in the basal diet, micro capsuled OEO at a concentration of 1.2% in the basal diet, garlic powder at a concentration of 0.4% in the basal diet, and garlic powder at a concentration of 2% in the basal diet. The plots show median (line within box), 25th and 75th percentiles (box), and minimum and maximum values (whiskers).

The effects of OEO and garlic supplementation on the serum oxidative stress biomarkers are presented in Figure 1.

When control and ZnO were compared with supplemented groups, control and ZnO groups showed higher serum catalase activity than garlic at 2% (*p* < 0.01). Serum TEAC was significantly higher (*p* < 0.05) in the control group than in OEO and garlic at 0.4% but significantly lower in the ZnO group when compared to OEO at 1.2% (*p* < 0.05). Serum CUPRAC was higher in the control group than in garlic at 2% (*p* < 0.05) and lower in ZnO when compared to OEO (*p* < 0.001) and garlic (*p* < 0.05) at 0.4% concentrations.

When the different concentrations of the supplemented groups were compared, serum catalase and CUPRAC were higher in OEO at 0.4% than in OEO at 1.2% (*p* < 0.01). In addition, they were higher in garlic at 0.4% than in garlic at 2% (*p* < 0.01). The group receiving OEO at 0.4% showed lower TEAC than OEO at 1.2%. TEAC was also lower in garlic at 0.4% than in garlic at 2% (*p* < 0.01).

Serum TOS, AOPP, and H_2_O_2_ were not different (*p* < 0.05) between the groups (Figure 1). Uric acid was below the detection limit in serum for all groups (data not shown).

**Figure 2:**
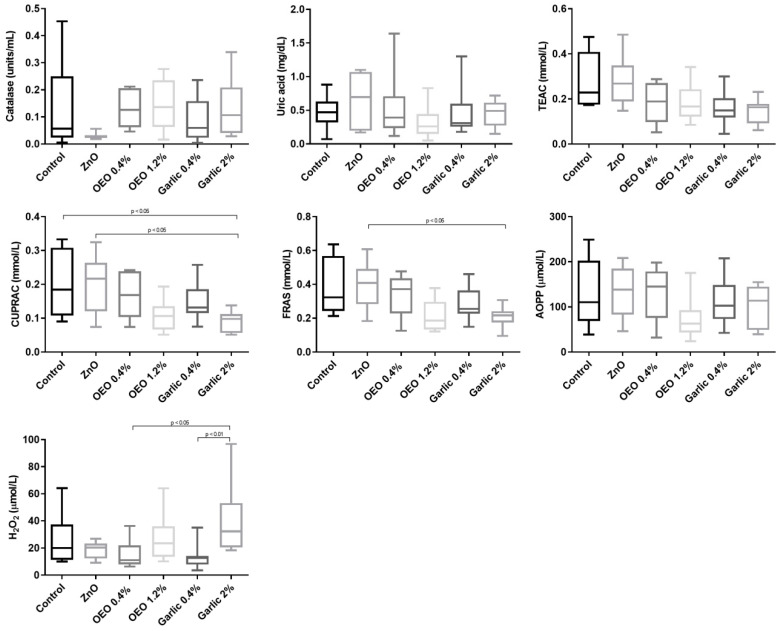
Results for salivary catalase, uric acid, Trolox equivalent antioxidant capacity (TEAC), cupric reducing antioxidant capacity (CUPRAC), ferric reducing ability of saliva (FRAS), advanced oxidation protein products (AOPP), and hydrogen peroxide (H_2_O_2_) in piglets supplemented with ZnO, micro capsuled OEO at a concentration of 0.4% in the basal diet, micro capsuled OEO at a concentration of 1.2% in the basal diet, garlic powder at a concentration of 0.4% in the basal diet, and garlic powder at a concentration of 2% in the basal diet. The plots show median (line within box), 25th and 75th percentiles (box), and minimum and maximum values (whiskers).

The effects of supplementation diets on salivary biomarkers of oxidative stress are shown in Figure 2. When control and ZnO were compared to supplemented groups, the control and ZnO groups presented higher (*p* < 0.05) salivary CUPRAC than the group supplemented with garlic at 2%. In addition, the ZnO group showed higher salivary FRAS when compared to the garlic at 2% group.

When the different concentrations of the supplemented groups were compared, garlic at 2% presented higher salivary H_2_O_2_ than garlic at 0.4% (*p* < 0.01). Salivary TEAC, AOPP, uric acid, and catalase showed no differences (*p* < 0.05) between the groups (Figure 2).

### 3.2. Salivary Cortisol

**Figure 3:**
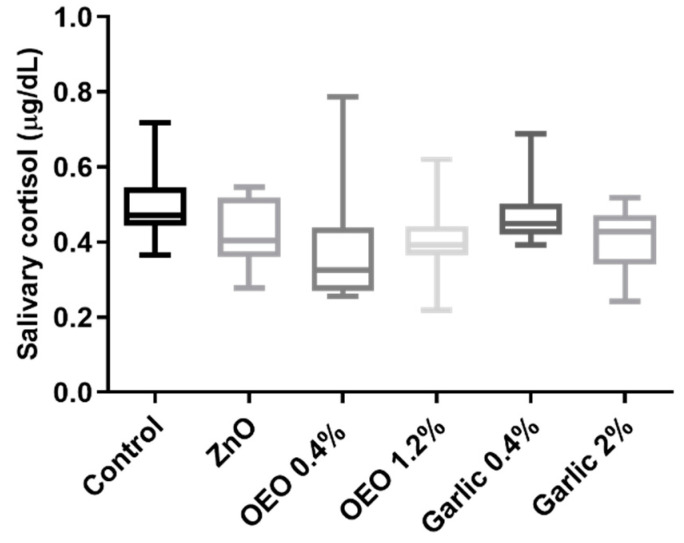
Results for salivary cortisol in piglets supplemented with ZnO, micro capsuled OEO at a concentration of 0.4% in the basal diet, micro capsuled OEO at a concentration of 1.2% in the basal diet, garlic powder at a concentration of 0.4% in the basal diet, and garlic powder at a concentration of 2% in the basal diet. The plots show median (line within box), 25th and 75th percentiles (box), and minimum and maximum values (whiskers).

The cortisol results are presented in Figure 3. They were not different between the groups (*p* > 0.05).

### 3.3. Serum CRP

**Figure 4:**
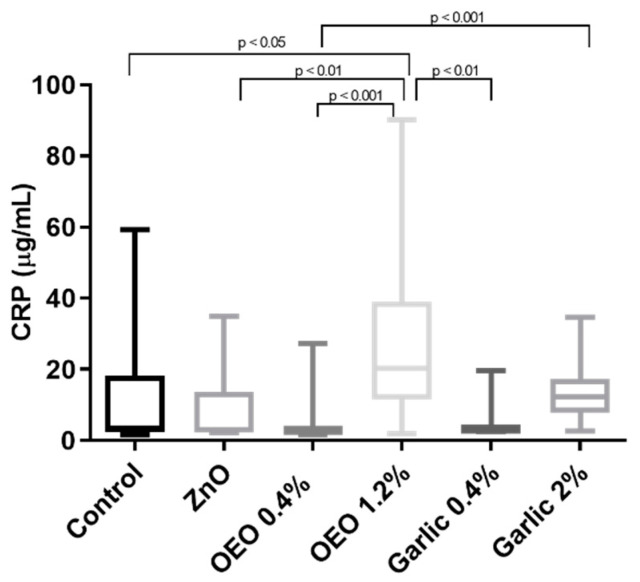
Results for serum C-reactive protein (CRP) in piglets supplemented with ZnO, micro capsuled OEO at a concentration of 0.4% in the basal diet, micro capsuled OEO at a concentration of 1.2% in the basal diet, garlic powder at a concentration of 0.4% in the basal diet, and garlic powder at a concentration of 2% in the basal diet. The plots show median (line within box), 25th and 75th percentiles (box), and minimum and maximum values (whiskers).

Higher CRP concentrations were observed in OEO at 1.2% when compared with all groups (*p* < 0.05) except for garlic at 2% (Figure 4). In addition, CRP was significantly increased in garlic at 2% when compared with OEO at 0.4% (*p* < 0.001; see Figure 4).

### 3.4. Correlations Between Serum and Saliva

CUPRAC and TEAC showed weak but significantly positive (*r* = 0.36, *p* < 0.01) and negative (*r* = −0.30, *p* < 0.01) correlations, respectively, between serum and saliva values.

### 3.5. Productive Parameters

The values of ADG (Table 5) in the OEO at low concentration group (mean ± SD: 0.3511 ± 0.02192 kg/day) were significantly higher than the values for the control (mean ± SD: 0.2650 ± 0.06253 kg/day; *p* = 0.001) and ZnO groups (mean ± SD: 0.2633 ± 0.03670 kg/day; *p* = 0.001).

Garlic at low concentration (mean ± SD: 0.3423 ± 0.03205 kg/day) led to a higher ADG values than in control (mean ± SD: 0.2650 ± 0.06253 kg/day; *p* = 0.001) and ZnO groups (mean ± SD: 0.2633 ± 0.3670 kg/day; *p* = 0.005) and did not show any significant difference with the OEO at low concentration group (*p* = 0.996) or with the groups with high concentrations of OEO and garlic (*p* = 0.615 and *p* = 0.126, respectively). Both groups with high concentrations of OEO and garlic did not show statistically significant differences with the rest of the groups.

Regarding FBW (Table 5), the OEO at low concentration (mean ± SD: 22.7013 ± 1.155 kg) and the garlic at low concentration groups (mean ± SD: 22.5238 ± 1.570 kg) showed higher values than the control (mean ± SD: 18.6450 ± 3.064 kg; *p* = 0.002 and *p* = 0.004, respectively) and ZnO groups (mean ± SD: 18.4933 ± 1.798 kg; *p* = 0.002 and *p* = 0.003 respectively). Both groups with high concentrations of OEO and garlic did not show statistically significant differences with the rest of the groups.

Regarding FCR and AFI, there were no significant differences between the groups (*p* = 0.069 and *p* = 0.149, respectively; Table 5).

## 4. Discussion

In this study, purple garlic powder and micro capsuled OEO were given at different concentrations as supplements to piglets during the weaning period, and their effects in biomarkers of oxidative status, stress, and inflammation, as well as in productive parameters, were evaluated. 

The group supplemented with higher doses of OEO showed higher serum TEAC when compared to OEO and garlic at lower doses and ZnO groups. These increases could be related to the inflammation produced when higher concentrations of OEO were given, as evidenced by the increased CRP concentrations observed in this group. It could be hypothesized that the antioxidants represented by TEAC increase their concentrations to counteract adverse reactions related to inflammation [68]. The tendency of FRAP to also increase in the group receiving OEO at high doses, when compared to low doses of the same supplement, would support our hypothesis. In contrast, catalase, an endogenous antioxidant, was significantly diminished in the groups receiving higher doses of OEO and garlic in comparison with their respective lower doses. Catalase is one of the main parameters involved in the cellular defence against free radicals, and its decrease could be related to the inflammation caused by the higher concentrations of the supplements given. Previous research showed that weaned piglets challenged with lipopolysaccharide showed lower catalase activity [69], which could be in line with the finds of this study.

On the other hand, CUPRAC increased its concentration when low doses of OEO and garlic were given, when compared with the groups that received higher doses of the same products. This different behaviour of CUPRAC, compared to TEAC and FRAP, could be related to the different components that are in CUPRAC, such as thiol, β-carotene and α-tocopherol, that, for example, in the case of β-carotene and α-tocopherol, could have proven to be beneficial for health and reproduction [70,71,72]. Therefore, the increase in CUPRAC with OEO and garlic supplement at low doses could indicate an improvement in the antioxidant status of the animals.

Regarding salivary biomarkers, piglets supplemented with high doses of garlic exhibited decreased antioxidants biomarkers, such as CUPRAC, in comparison with ZnO. On the other hand, the same compound in high doses led to an increase in oxidant biomarkers such as H_2_O_2_ when compared with low concentrations of both OEO and garlic. This increase in oxidant compounds in saliva would be in line with the changes of biomarkers of oxidative stress in serum at higher doses of garlic powders, which could be due to the toxic effects described in farm animals after the consumption of garlic or onions at high doses, including oxidative damage to erythrocytes [73].

Based on the results of this trial, high concentrations of OEO or garlic power will lead to a worse situation in oxidative status compared to the use of lower concentrations of these supplements. This has been previously reported with some oregano compounds such as carvacrol and thymol since, at high doses, they induce DNA damage in human lymphocytes. In contrast, lower doses of thymol and carvacrol, which possess useful antioxidant properties, significantly reduced oxidative DNA damage in human lymphocytes [37]. 

The lack of significant changes in salivary cortisol between the different groups could indicate that the different supplements tested in this study do not produce a significant activation of the hypothalamic-pituitary-adrenal (HPA) axis in any group of piglets and, therefore, they do not induce a stress response. Although in this research, we did not evaluate the response of the different groups against a model of stress, serum cortisol concentrations after a model of transport were markedly lower in those animals receiving supplementation with OEO than in those fed with a control diet [31]. Considering this, an interesting line of future research would be the potential of using these supplements at low doses to reduce activation of the HPA axis in stressful situations.

In our study, higher serum CRP was observed when high doses of OEO were given. From a toxicological point of view, it has been reported that excessive oral doses of oregano can cause gastrointestinal irritation [74], which could explain the higher levels of serum CRP and inflammation observed in the group that received OEO at high concentrations. Conversely, the groups supplemented with low concentrations of OEO and garlic powder presented lower serum values of CRP, which is in line with the described anti-inflammatory effects of oregano and garlic in pigs [41,75]. 

Regarding productive parameters, OEO at low doses produced higher results of ADG in postweaning piglets, showing significantly higher values compared to the rest of the groups except for garlic at low concentration. Higher FBW values were also obtained for OEO and garlic at low doses, which were significantly higher than the control and ZnO FBW values. These results would be in line with the better results of the oxidative stress biomarkers, cortisol and CRP in the groups supplemented with low doses of OEO and garlic. Positive effects on ADG and average daily feed intake (ADFI) when low doses of fermented garlic were given to piglets have been described [76,77]. In addition, improved body weight due to supplementation of garlic or garlic-derived products in pigs has also been reported [39,41,78].

## 5. Conclusions

It could be concluded that doses of OEO and garlic powder at 0.4% did not lead to inflammation, stress, or negative changes in oxidative biomarkers in piglets during the postweaning period. In addition, they gave better productive performance than the control and ZnO diets. However, high doses of those supplements were shown to be ineffective and could negatively affect the animals. Therefore, our results highlight the importance of the dose used when OEO or garlic are supplemented to piglets.

## Figures and Tables

**Table 1:** Bioactive components of the oregano essential oil (OEO).

Components	%
α-Pinene	0.84
α-Thuyene	1.10
Camphene	0.07
β-Pinene	0.23
β-Mircene	1.56
α-Phellandrene	0.22
α-Terpinene	1.25
Limonene	0.31
1,8-Cineole+ β-Phellandrene	0.30
γ-Terpinene	5.20
3-Octonone	0.11
p-Cymene	5.99
Terpinolene	0.12
1-Octen-3-ol	0.26
(E)-Thuyanol	0.10
Linalool	1.73
(Z)-Thuyanol	0.05
1-Octanol	0.02
Terpinene-4-ol	0.79
β-Caryophyllene	2.59
α-Humulene	0.12
α-Terpineol	0.16
Borneol	0.22
β-Bisabolene	0.23
Caryophellene oxyde	0.14
Thymol	4.10
Carvacrol Isomere	0.05
Carvacrol	70.32
Total	98.19

**Table 2:** Chemical composition and amino acid composition of the purple garlic powder.

Analytical Components	Quantity
Protein (%)	3.9
Carbohydrates (%)	15.4
Sugars (%)	1.4
Crude Fibre (%)	0.7
Crude Fat (%)	0.2
Crude Ash (%)	35.4
Humidity (%)	44.4
Inuline (%)	8.1
Energy (kJ/kg)	341
**Aminoacids (g/kg)**	
Methionine	0.5
Lysine	2.1
Isoleucine	0.9
Leucine	1.5
Valine	1.4
Phenylalanine	1.1
Tryptophan	0.4
Threonine	1.1
Histidine	0.6
Arginine	8.8
**Minerals (mg/kg)**	
Calcium	1100
Phosphorus	1060
Potassium	3500
Sodium	7247
Copper	1.6
Cobalt	0.12
Iron	20
Manganese	13.2
Selenium	0.2
Zinc	6.3
Sulfur	6000
**Vitamins**	
Thiamine (mg/kg)	2.6
Niacin (mg/kg)	17
Ascorbic acid (mg/kg)	116.5
Cholecalciferol (µg)	4
Riboflavin (mg/kg)	0.3
Cyanocobalamin (µg)	5
Retinol (mg/kg)	0.23
Tocopherol (mg/kg)	3

**Table 3:** Composition of the control diet.

Ingredients Prestarter (g/kg)	Prestarter	Starter
Maize	299.8	
Wheat	200	
Soybean	183.5	
Soybean meal	135	
Barley	80	
Whey	75	
Vitamins and oligoelements premix	25	
Lysine	0.75	
Threonine	0.6	
Tryptophan	0.2	
Methionine	0.15	
**Ingredients Starter (g/kg)**		
Maize		329.5
Barley		250
Wheat		170
Soybean		129
Soybean meal		61
Vitamins and oligoelements premix		25
Soy husk		10
Liquid lysine		7.5
Monocalcic phosphate		5.5
Lard		5
Salt		4
Calcium carbonate		3.5
**Vitamins**		
Vitamin A UI/kg	6500	6500
Vitamin D3 UI/kg	1500	1500
Vitamin E UI/kg	92	92
Coline chloride mg/kg	1356	1356
**Oligoelements (mg/kg)**		
Fe	120	120
Mn	30	30
Zn	110	110
Se	0.20	0.20
I	0.8	0.8
Cu	80	80
**Analytical Components (%)**		
Crude protein	16.0	16.0
Crude fibre	2.7	4.0
Crude fat	3.8	3.0
Crude ash	6.3	4.3
Calcium	0.85	0.72
Phosphorus	0.67	0.50
Sodium	0.26	0.25
Metionine	0.49	0.44
Lisine	1.18	1.09

**Table 4:** Bodyweight at the beginning of the trial ^1^.

Group	Mean (kg)	SEM (kg)
Control	5.66	0.072
ZnO	5.59	0.070
OEO 0.4%	5.49	0.090
OEO 1.2%	5.87	0.061
Garlic 0.4%	5.68	0.082
Garlic 2%	5.67	0.174

^1^ Data are the means of 10 replicates of 5 pigs each per treatment.

**Table 5:** Values of average daily gain (ADG) and feed conversion ratio (FCR) for the different groups ^1,2^.

Group	ADG		FCR		Feed Intake		FBW	
	Mean	SEM	Mean	SEM	Mean	SEM	Mean	SEM
Control	0.265 ^a^	0.025	2.120	0.187	26.763	1.839	18.645 ^a^	1.251
ZnO	0.263 ^a^	0.014	1.811	0.091	23.150	0.992	18.493 ^a^	0.734
OEO 0.4%	0.351 ^c^	0.007	1.456	0.089	24.974	1.373	22.701 ^b^	0.408
OEO 1.2%	0.307 ^abc^	0.007	1.787	0.064	26.903	0.877	20.937 ^ab^	0.368
Garlic 0.4%	0.342 ^bc^	0.011	1.876	0.217	31.338	3.442	22.523 ^b^	0.555
Garlic 2%	0.285 ^ab^	0.009	1.985	0.074	27.710	1.275	19.635 ^ab^	0.469

^1^ The different superscripts (a, b, c) indicate statistically significant differences between the groups (*p* ≤ 0.05). ^2^ Data are the means of 10 replicates of 5 pigs each per treatment.
